# Short-term outcomes of thoracoscopic versus open lobectomy for congenital lung malformations

**DOI:** 10.1007/s00383-023-05445-7

**Published:** 2023-03-22

**Authors:** Steven L. Raymond, Marla A. Sacks, Asra Hashmi, Jason O. Robertson, Donald Moores, Edward P. Tagge, Andrei Radulescu, Saleem Islam, Faraz A. Khan

**Affiliations:** 1 Division of Pediatric Surgery, Department of Surgery, Loma Linda University School of Medicine, 11175 Campus St, Suite 21111, Loma Linda, CA USA; 2 Division of Plastic Surgery, Department of Surgery, Kaiser Permanente, San Jose, CA USA; 3 Department of Pediatric Surgery, Cleveland Clinic Children’s Hospital, Cleveland, OH USA; 4 Division of Pediatric Surgery, Department of Surgery, University of Florida College of Medicine, Gainesville, FL USA

**Keywords:** Minimally invasive surgery, Video-assisted thoracoscopic surgery, Pediatric, CLM, CPAM

## Abstract

**Purpose:**

Thoracoscopic and open approaches for the management of congenital lung malformations (CLM) has been debated. The aim of this study is to compare 30-day outcomes for non-emergent lobectomies in children.

**Methods:**

The National Surgical Quality Improvement Program-Pediatric database was queried for patients undergoing CLM resection from 2013 to 2020. Outcomes were compared by operative technique in an intention-to-treat model and then propensity matched.

**Results:**

2157 patients met inclusion criteria and underwent non-emergent pulmonary lobectomy for CLM. The intended operative approach was thoracoscopic in 57.7% of patients. Patients in the open group compared to the thoracoscopic were more likely to be born premature, have chronic lung disease, require preoperative oxygen support, and be ventilator dependent. After propensity matching, there was no statistically significant difference in 30-day mortality, unplanned readmission, and other complications between the thoracoscopic and open groups. Thoracoscopic approach was associated with a shorter length of stay. The proportion of cases approached via thoracoscopy increased over time from 48.8% in 2013 to 69.9% in 2020.

**Conclusions:**

This large multicenter retrospective matched analysis demonstrates thoracoscopic lobectomy in children has similar favorable 30-day outcomes and shorter length of stay for the non-emergent management of CLM, compared to open thoracotomy.

**Level of evidence:**

Level III.

## Introduction

Congenital lung malformations (CLM) encompass several distinct diagnoses, including congenital lobar emphysema (CLE), intrapulmonary bronchogenic cysts, bronchopulmonary sequestration (BPS), and congenital pulmonary airway malformation (CPAM) [1]. Due to overlapping clinical, radiographic, and pathological features, and the existence of hybrid lesions, these diagnoses are frequently considered together. These lesions are rare but remain the most common indication for pulmonary lobectomy in children [2].

The majority of neonates with CLM are asymptomatic at birth, with less than 20% exhibiting significant symptoms [3, 4]. A proportion of symptomatic neonates are prone to need significant ventilator support and may require extracorporeal membrane oxygenation. If emergent surgery is needed, it can be associated with significant morbidity (28%) and mortality (7.5%) [4]. Some neonates that were asymptomatic at birth will develop symptoms as early as seven months of age including mechanical complications such as pneumothorax secondary to cyst rupture [3, 5]. However, the most common symptoms outside of the neonatal period are recurrent pulmonary infections, which occur at a rate of 10–30% within the first year of life [5].

CPAMs have a pre-malignant potential, although the risk of malignant transformation and the exact prevalence of this association remains unclear. There are reports of pleuropulmonary blastoma (PPB) and bronchioloalveolar carcinoma arising in the setting of pre-existing CPAM suggesting the malignant transformation of the underlying CPAM [3, 6, 7]. Additionally, there are no clinical or radiographical findings that clearly differentiate CPAMs from PPBs.

The risk of recurrent pulmonary infections and the possible association with malignant tumor transformation are typically what drive pediatric surgeons to recommend elective surgery. Given this unique situation where an otherwise seemingly healthy child is being offered a major elective surgical intervention, it becomes imperative to have high-quality evidence guiding surgical treatment. Open thoracotomy was traditionally considered the standard approach for the surgical treatment of CLM. Beginning in 2003, several reports described the safety and efficacy of minimally invasive lung resections [2, 4, 5, 8]. Polites et al. reported a considerable increase in the application of minimally invasive approaches to pulmonary lobectomy during the last decade [9]. While the thoracoscopic approach now appears to be the most common approach to elective pulmonary lobectomy, there remains marked variation in the application of this technique between institutions [10]. The outcomes of minimally invasive pulmonary resections have been heterogenous due to the relative rarity of this anomaly, marked differences in institutional experience, and a steep learning curve to achieve technical proficiency due to the small working space, anatomical variations, and the delicate nature of the pulmonary vascular structures [11]. Comparative studies between the two approaches have been limited by small sample sizes and marked heterogeneity in patient characteristics.

The aims of this study were to assess if outcomes for pediatric patients with CLMs are independently associated with the surgical approach for non-emergent pulmonary lobectomy and if outcomes for the thoracoscopic approach have changed over time. To accomplish these aims, we evaluated the National Surgical Quality Improvement Program-Pediatric (NSQIP-P) database using a propensity-matched methodology.

## Methods

### Study design

Data were collected from the American College of Surgeons (ACS) NSQIP-P Participant Use Data File for the years 2013–2020. The NSQIP-P is a large validated and risk-adjusted dataset containing 812,852 pediatric surgery cases prospectively collected by 141 hospitals. Patients included in this study were < 18 years of age at the time of surgery and had a diagnosis of CLM. Cases were identified using the International Classification of Disease, Ninth Revision (ICD-9) and ICD-10 diagnoses codes: CPAM (ICD-9: 748.4, ICD-10: Q33.0), BPS (ICD-9: 748.5, ICD-10: Q33.2), CLE (ICD-9: 770.2, 492, 492.8, 518.1, ICD-10: P25.0, P25.8), and other congenital lung lesion (ICD-9: 748.6, 748.69, 748.8, 718.89, 793.19, ICD-10: Q33.8). The foci of this study are the outcomes of non-emergent lobectomies in pediatric patients, as such patients less than 30 days old at the time of surgery, emergent resections, and wedge resections were excluded.

### Variables, outcomes, and definitions

The ACS NSQIP-P collects data on over 150 variables including preoperative risk factors, intraoperative variables, and 30-day postoperative mortality and morbidity outcomes. Demographic data included age, sex, and weight. Comorbidities included a history of prematurity, bronchopulmonary dysplasia/chronic lung disease, preoperative supplemental oxygen requirement, cardiac risk factors, and ventilator dependence. American Society for Anesthesiologists (ASA) class and case status (elective, urgent, emergent) were also recorded. The primary clinical outcome was 30-day postoperative mortality and morbidity including blood transfusion, mechanical ventilation for greater than 48-h, reoperation, pneumonia, and unplanned intubation. Secondary clinical outcomes included total operation time, surgical time, length of hospital stay, and unplanned readmission within 30 days from the operation. Surgical time was calculated as total operative time minus the sum of the duration from anesthesia start to surgery start and duration from surgery stop to anesthesia stop.

### Data analyses

Data analyses were performed using the R software package (The R Foundation for Statistical Computing, V.4.1.3). Data are presented as frequency and percentage for categorical variables and as the median and interquartile range (IQR) for continuous variables. Continuous variables were analyzed using student’s *t* test for parametric data, or the Mann–Whitney *U* test for non-parametric data. Categorical variables were analyzed using proportional statistics (Fischer’s Exact Test). Propensity score matching was used to minimize selection bias when comparing outcomes between groups. Thoracoscopic and open groups were matched 1:1 based on a set of variables (age, ASA class, preoperative oxygen requirement, case status, chronic lung disease, and ventilator dependence) that could otherwise confound comparisons between the groups. To analyze changes in the outcomes of a thoracoscopic approach over time, thoracoscopic patients alone were compared between the era within which the operation was performed (2013–2016 or 2017–2020) and matched 1:1 based on the same above-listed set of variables. To analyze independent variables associated with a probability of conversion from thoracoscopic to open surgery, multivariate logistic regression models were used with results presented as odds ratio and 95% confidence intervals (CI). All significance tests were two-sided, with *p* values less than 0.05 considered statistically significant.

## Results

### Patient demographics and management

2157 patients with congenital lung malformations met the study criteria, and overall cohort demographics are provided in Table 1. There was a slight male predominance (55.2%) and the median age at surgery was 271 days [IQR 174, 642]. Lung resections were performed for 1,358 CPAMs (63.0%), 48 BPSs (2.2%), and 3 CLEs (3.4%). The remaining cases were hybrid or unspecified CLM. 57.7% (1244) were managed with an intention of thoracic resection and 42.3% (913) via an open approach. 8.4% (181) required conversion from thoracoscopic to open and these patients were analyzed in the thoracoscopic group. Patients in the open group compared to the thoracoscopic group were more likely to be premature (*p* = 0.013), have chronic lung disease (*p* = 0.005), require oxygen support prior to surgery (*p* < 0.0001), and be ventilatory dependent (*p* < 0.0001).

**Table 1:** Demographics and preoperative characteristics

	All patients (*n* = 2157)	Thoracoscopic (*n* = 1244, 57.7%)	Open (*n* = 913, 42.3%)	*p* value*
Age (days), median [IQR]	271 [174, 642]	260 [171, 485]	295 [175, 1098]	0.0006
Age group, *n* (%)				
< 30 days	0 (0)	0 (0)	0 (0)	NA
30 d-6 months	594 (27.5)	352 (28.3)	242 (26.5)	0.380
6–12 months	763 (35.4)	484 (38.9)	279 (30.6)	< 0.0001
1–2 years	369 (17.1)	207 (16.6)	162 (17.7)	0.525
3–5 years	111 (5.1)	56 (4.5)	55 (6.0)	0.116
6–12 years	145 (6.7)	66 (5.3)	79 (8.7)	0.003
> 12 years	175 (8.1)	79 (6.4)	96 (10.5)	0.0006
Sex, *n* (%)				
Female	967 (44.8)	545 (43.8)	422 (46.2)	0.274
Male	1190 (55.2)	699 (56.2)	491 (53.8)	
Weight (kg), median [IQR]	19.1 [15.6, 26.2]	18.7 [15.6, 24.3]	19.7 [15.6, 31.2]	0.005
Comorbidities, *n* (%)				
Premature	140 (6.9)	67 (5.7)	73 (8.5)	0.013
Lung disease	209 (9.7)	101 (8.1)	108 (11.8)	0.005
Preop O_2_ requirement	78 (3.6)	20 (1.6)	58 (6.4)	< 0.0001
Cardiac risk factors	297 (13.8)	159 (12.8)	138 (15.1)	0.129
Ventilator dependence	38 (1.8)	5 (0.4)	33 (3.6)	< 0.0001
ASA class, *n* (%)				
I	92 (4.3)	54 (4.3)	38 (4.2)	< 0.0001
II	1217 (56.4)	750 (60.3)	467 (51.2)	
III	787 (36.5)	427 (34.3)	360 (39.5)	
IV	60 (2.8)	13 (1.0)	47 (5.2)	
Case status, *n* (%)				
Elective	2105 (97.6)	1225 (98.5)	880 (96.4)	0.003
Urgent	52 (2.4)	19 (1.5)	33 (3.6)	
Wound class, *n* (%)				
Clean	828 (38.4)	482 (38.7)	346 (37.9)	0.446
Clean contaminated	1258 (58.3)	726 (58.4)	532 (58.3)	
Contaminated	34 (1.6)	15 (1.2)	19 (2.1)	
Dirty/infected	37 (1.7)	21 (1.7)	16 (1.8)	

*p value corresponds to the comparison of thoracoscopic and open groups

### Clinical outcomes

Clinical outcomes for the study cohort are listed in Table 2. The 30-day survival was 99.7%, and the median hospital length of stay was 3 days. Overall, the 30-day composite adverse event (blood transfusion, mechanical ventilation for greater than 48-h, reoperation, pneumonia, unplanned intubation, unplanned readmission, morality) rate was 15.3%. In the unmatched comparison, length of stay (*p* < 0.0001), blood transfusion (*p* = 0.014), mechanical ventilation for greater than 48 h (*p* < 0.0001), and mortality (*p* = 0.006) were all increased in the open group.

**Table 2:** Unmatched comparison of clinical outcomes based on surgical approach

	All patients (*n* = 2157)	Thoracoscopic (*n* = 1244, 57.7%)	Open (*n* = 913, 42.3%)	*p* value*
Surgical time (min), median [IQR]	92 [50, 148]	100 [57, 154]	81 [42, 141]	< 0.0001
Total operative time (min), median [IQR]	172 [124, 231]	182 [131, 236]	160 [115,223]	< 0.0001
LOS (days), median [IQR]	3 [2, 5]	3 [2, 4]	3 [2, 6]	< 0.0001
Postop complications, *n* (%)				
Transfusion	133 (6.2)	63 (5.1)	70 (7.7)	0.014
Ventilation > 48 h	58 (2.7)	18 (1.4)	40 (4.4)	< 0.0001
Reoperation	106 (4.9)	62 (5.0)	44 (4.8)	0.920
Pneumonia	26 (1.2)	17 (1.4)	9 (1.0)	0.550
Unplanned intubation	25 (1.2)	15 (1.2)	10 (1.1)	0.842
Unplanned readmission, *n* (%)	119 (5.5)	68 (5.5)	51 (5.6)	0.924
Mortality, *n* (%)	6 (0.3)	0 (0)	6 (0.7)	0.006
Any complication, unplanned readmission, or mortality, *n* (%)	331 (15.3)	176 (14.1)	155 (17.0)	0.079

*p value corresponds to comparison of thoracoscopic and open groups

**Fig. 1:**
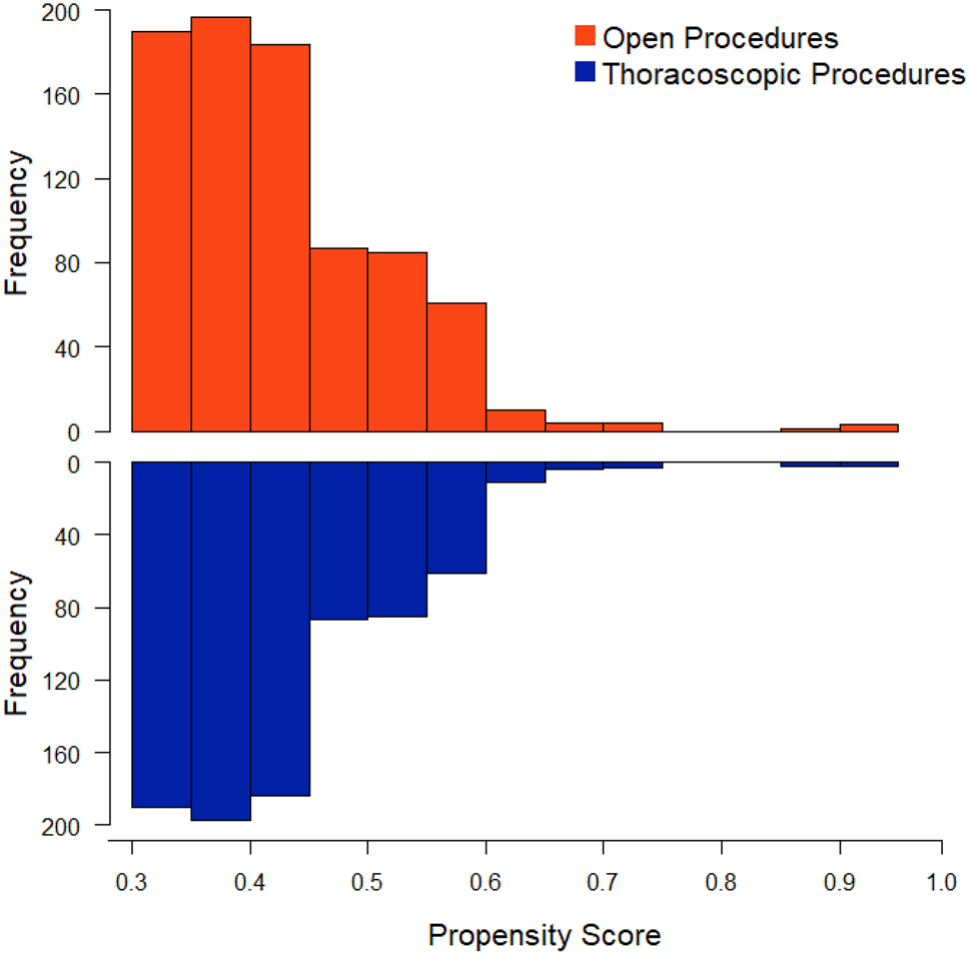
Propsentiy score distribution graph comparing propsentiy scores between matched thorasocopic approach and the open approach

The propensity score-matched cohort was comprised of 1652 patients (Fig. 1). 826 in the thoracoscopic group (50%) and 826 in the open group (50%) (Table 3). Previously observed covariate imbalances between treatment groups (prematurity, preoperative oxygen requirement, ventilation dependence) were alleviated after matching. In the propensity-matched cohort, the overall survival was 99.9% and the 30-day composite adverse event rate was 13.6%. After matching, there were no statistically significant differences in 30-day mortality, unplanned readmission, and other complications. Surgical time remained longer for the thoracoscopic approach compared to the open approach (103 min vs. 81 min; *p* = 0.0002). The length of hospital stay was shorter for the thoracoscopic group (median 3 days [IQR 2, 4]) compared to the open group (median 3 days [IQR 2, 6]) (*p* = 0.046).

**Table 3:** Matched comparison of clinical outcomes based on surgical approach (patients matched on age, ASA class, oxygen requirement, case status, chronic lung disease, cardiac risk factors, and ventilator dependence)

	Thoracoscopic (*n* = 826, 50.0%)	Open (*n* = 826, 50.0%)	*p* value*
Age (days), median [IQR]	286 [182, 821]	288 [176, 758]	0.494
Weight (kg), median [IQR]	19.6 [16.0, 28.6]	19.6 [15.6, 28.2]	0.542
Comorbidities, *n* (%)			
Premature	43 (5.2)	50 (6.1)	0.592
Lung dx	73 (8.8)	85 (10.3)	0.018
Preop O_2_ requirement	19 (2.3)	22 (2.7)	0.200
Cardiac risk factors	96 (11.6)	95 (11.5)	0.641
Ventilator dependence	4 (0.5)	5 (0.6)	0.741
ASA Class, *n* (%)			
I	37 (4.5)	38 (4.6)	0.596
II	475 (57.5)	465 (56.3)	
III	303 (36.7)	304 (36.8)	
IV	11 (1.3)	19 (2.3)	
Case status, *n* (%)			
Elective	808 (97.8)	811 (98.2)	0.484
Urgent	18 (2.2)	15 (1.8)	
Wound class, *n* (%)			
Clean	326 (39.5)	311 (37.7)	0.518
Clean contaminated	469 (56.8)	492 (59.6)	
Contaminated	12 (1.5)	13 (1.6)	
Dirty	19 (2.3)	10 (1.2)	
Total operative time (min), median [IQR]	183 [128, 240]	160 [115, 220]	0.001
Surgical time (min), median [IQR]	103 [61, 158]	81 [42, 141]	0.0002
LOS (days), median [IQR]	3 [2, 4]	3 [2, 5]	0.046
Postop complications, *n* (%)			
Transfusion	45 (5.4)	50 (6.1)	0.605
Ventilation > 48 h	16 (1.9)	14 (1.7)	0.706
Reoperation	41 (5.0)	33 (4.0)	0.354
Pneumonia	13 (1.6)	6 (0.7)	0.117
Unplanned intubation	11 (1.3)	5 (0.6)	0.144
Unplanned readmission, *n* (%)	36 (4.4)	45 (5.4)	0.312
Mortality, *n* (%)	0 (0)	2 (0.2)	0.511
Any complication, unplanned readmission or mortality, *n* (%)	113 (13.7)	111 (13.4)	0.887

*p value corresponds to comparison of thoracoscopic and open groups

### Thoracoscopic approach over time

In 2013, 48.8% of cases (59 of 121) were approached as a thoracoscopic resection. In comparison, 69.9% of cases (172 of 246) were approached via thoracoscopic resection in 2020. After propensity score-matching for a thoracoscopic approach based on the era when the operation was performed, there were 576 patients—285 in the 2013–2016 group (49.5%) and 291 in the 2017–2020 group (50.5%) (Table 4). There were no mortalities in this cohort. There was no statistically significant difference in 30-day unplanned readmission or other complications. The median surgical time was statistically similar between eras with 95 min required for thoracoscopic resections in 2013–2016 compared to 102 min for thoracoscopic resections in 2017–2020 (*p* = 0.519). The hospital length of stay was also similar between the two eras (2 days vs. 2 days, *p* = 0.412).

**Table 4:** Matched comparison of clinical outcomes based on era for thoracoscopic resections (patient matched on age, ASA class, oxygen requirement, case status, chronic lung disease, cardiac risk factors, and ventilator dependence)

	2013–2016 (*n* = 285, 49.5%)	2017–2020 (*n* = 291, 50.5%)	*p* value*
Surgical time (min), median [IQR]	95 [57, 150]	102 [62, 153]	0.519
Total operative time (min), median	170	182	0.355
LOS (day), median	2	2	0.412
Postop complications, *n* (%)			
Transfusion	13 (4.6)	9 (3.1)	0.392
Ventilation > 48 h	3 (1.1)	1 (0.3)	0.369
Reoperation	15 (5.3)	15 (5.2)	1.000
Pneumonia	5 (1.8)	4 (1.4)	0.750
Unplanned intubation	5 (1.8)	3 (1.0)	0.501
Unplanned readmission, *n* (%)	11 (3.9)	16 (5.5)	0.432
Mortality, *n* (%)	0 (0)	0 (0)	NA
Any complication, unplanned readmission or mortality, *n* (%)	37 (13.0)	36 (12.4)	0.901

*p value corresponds to comparison of 2013–2016 and 2017–2020 groups

### Factors associated with conversion from thoracoscopic to open surgery

Overall, 14.6% thoracoscopic cases were converted to open. Using multivariate logistic regression models, chronic lung disease was independently associated with the probability of conversion to open surgery (OR 2.1, 95% CI 1.22–3.57, *p* = 0.006). The year the operation was performed, patient age, weight, sex, ASA class, wound class, and other comorbidities were not independently associated with a probability of conversion.

## Discussion

Pediatric patients with congenital lung malformations undergoing non-emergent thoracoscopic lobectomies have similar favorable 30-day clinical outcomes, compared to non-emergent open thoracotomy. Additionally, the patient approached via thoracoscopy had a shorter length of stay. To our knowledge, this is the first study to use propensity-matched methodology to compare thoracoscopic and open techniques in the management of congenital lung malformations.

The findings reported here agree with a 2012 metanalysis of six retrospective studies which found no significant difference in overall complication rates between techniques [12]. In the study herein, patients had a median postoperative length of stay of three days which was statistically less for thoracoscopic approach compared to the open approach based on unmatched and matched analyses. This is consistent with a multi-institution European study of 102 patients undergoing thoracoscopic resection for congenital lung malformations which reported a median length of stay of 3.7 days [13]. This supports that thoracoscopic surgery is as safe as open approaches and leads to a shorter length of stay.

The proportion of cases performed via thoracoscopy increased over time from 48.8% in 2013 to 69.9% in 2020. Previously, a database study of 1120 patients ≤ 20 years old undergoing resection of congenital lung malformations demonstrated that utilization of a thoracoscopic approach increased from 32.2% in 2008 to 48.2% in 2012 [9]. Based on the data herein, clinical outcomes for thoracoscopic lobectomy did not change over time. Likewise, median operating time and conversion rate to open surgery did not change between eras. Chronic lung disease was the only variable independently associated with a probability of conversion to open surgery. This may be due to patients with chronic lung disease having a decreased physiologic tolerance of single lung ventilation and carbon dioxide insufflation, which are typically employed during a thoracoscopic approach. Clark et al. reported an overall 21.7% conversion rate based on a single institution's 10-year experience [14]. The majority of cases were converted secondary to an inability to tolerate single lung ventilation and the presence of intrathoracic adhesions. Of note, their conversion rate decreased over the study period, from 66.7% in 2010 to 0% in 2020 with an associated increased use in thoracoscopic approach during the same period. Likewise, the multicenter European study published in 2021 reported no conversions to open and a median operative time of 92.2 min [13]. Here, the median surgical time for thoracoscopic resection was 100 min.

Though thoracoscopic and open surgery have similar short-term clinical outcomes, the long-term musculoskeletal and aesthetic complications of thoracotomy make minimally invasive surgery the favorable approach. Thoracotomy performed during childhood is associated with the development of clinically significant musculoskeletal abnormalities, including scoliosis with a rate of up to 62% [15–20]. In a 2009 comparison of children undergoing thoracoscopic surgery versus conventional thoracic surgery, the incidence of scoliosis was significantly lower in the thoracoscopic group (9% vs. 54%, *p* < 0.001) [20]. Additionally, scar assessment scores of patients undergoing thoracoscopic surgery were significantly better [20]. Taken together, these findings support advocacy for a minimally invasive approach.

Some have previously described non-operative management of congenital lung malformations; however, studies describing a non-operative approach are marred by limited clinical and appropriate imaging follow-up [21]. The vast majority (86%) of initially asymptomatic patients later develop symptoms [22]. Additionally, those managed without surgical resection still require cross-sectional imaging, which over time can lead to significant cumulative radiation exposure [21]. Moreover, some initially asymptomatic patients may require surgery on a more urgent basis if the decision for resection is delayed, and operations performed outside of the elective setting are associated with an increased risk of postoperative complications [9]. Herein, the authors have demonstrated that non-emergent resection is safe and can be completed thoracoscopically with good outcomes, minimal additional operative time, and a slightly reduced length of study. Postoperative mortality remains extremely rare, and reported complications are low.

There are potential limitations to the study that require mention. Despite the large cohort and use of propensity matching, the study was retrospective in design, and patients were not randomized to any particular management. Therefore, selection bias of patients to specific treatment could influence the observed outcomes. Additionally, analyses were limited to clinical variables available within the database; for example, excluded was congenital pulmonary airway malformation volume ratio (CVR) which is important in characterizing and risk stratifying CPAMs. The data were abstracted from deidentified medical centers. Given differences in institutional surgical volume, expertise, and equipment among medical centers, it is unknown how institutional factors may affect outcomes and whether comparable results can be expected at other medical centers. Likewise, it is not known from this study the impact of surgeon volume has on outcomes, and this requires further investigations. Despite the study limitations, the cohort size and the clinical nature of the data collected is a substantial source of strength and aids the growing body of literature on surgical approach for congenital lung malformations. Additionally, the use of propensity score matching is a strength and minimizes selection bias when comparing outcomes between groups. To date, this methodology had not been previously employed to investigate the surgical management of this congenital condition.

## Conclusions

This large multicenter, validated dataset demonstrates pulmonary resections of congenital lung malformations in children are safe. Using a matched propensity model, thoracoscopic and open surgical approaches had comparable 30-day outcomes for non-emergent lobectomies. Thoracoscopic approaches had a shorter length of stay. Nationally pediatric surgeons appear to be increasing their use of thoracoscopic techniques over time.

## Abbreviations

ACS: American college of surgeons
ASA: American society for anesthesiologists
BPS: Bronchopulmonary sequestration
CI: Confidence interval
CCAM: Congenital cystic adenomatoid malformation
CLE: Congenital lobar emphysema
CLM: Congenital lung malformations
CPAM: Congenital pulmonary airway malformation
CVR: Congenital pulmonary airway malformation volume ratio
ICD: International classification of disease
IQR: Interquartile range
NSQIP-P: National Surgical Quality Improvement Program-Pediatric
PPB: Pleuropulmonary blastoma
